# ASSETS, GAPS, AND OPPORTUNITIES FOR AGE INCLUSIVITY: THE AIDHE MODEL, THE ICCS MEASURE, AND FURTHER APPLICATIONS

**DOI:** 10.1093/geroni/igae098.1432

**Published:** 2024-12-31

**Authors:** Lauren Bowen, Nina Silverstein, Joann Montepare, Susan Whitbourne

**Affiliations:** University of Massachusetts Boston, Boston, Massachusetts, United States; University of Massachusetts Boston, Needham, Massachusetts, United States; Lasell University, Newton, Massachusetts, United States; University of Massachusetts Boston, Boston, Massachusetts, United States

## Abstract

The Age Inclusivity Domains of Higher Education (AIDHE) model was developed to encourage attention to age inclusivity across seven key domains of institutional function —Teaching and Learning, Student Affairs, Outreach and Engagement, Personnel, Physical Environment, Research, and Services and Resources—and across the experiences of age-diverse students, faculty, and staff. To assess age inclusivity across the seven domains, we (Silverstein et. al. 2022; 2024) developed and tested the Age-Friendly Inventory and Campus Climate Survey (ICCS), which documents the presence of age-friendly campus practices; perceptions of those practices by faculty, staff, and students; and match between practices and perceptions; further, the degree of match along with agreement in the value of age inclusivity predicts perceptions of age inclusivity by campus constituents. The AIDHE model and data collected using the ICCS have supported further research to expand age inclusivity efforts. In a multimethod study (Bowen et al., 2024), we identified key challenges and transformative, actionable strategies to advance age inclusivity in Teaching and Learning, Personnel, and Student Affairs domains. Data were collected from 2,273 ICCS open-ended question, as well as from 16 faculty, staff, and student focus groups, to identify both challenges and potential strategies. These challenges and strategies were reviewed over two rounds by Delphi expert panelists, representing higher education administrators and leaders. Final analysis balanced each strategy’s transformative potential (through impact and priority) with its actionable potential (through feasibility and likelihood of implementation), which ultimately yielded 23 recommended strategies intended to address key challenges of age inclusivity in higher education.

